# The Value of Hydrogen Peroxide in Neurosurgery and Its Pathophysiological Effects in Human and Animal Brain Tissues

**DOI:** 10.3390/ph18040533

**Published:** 2025-04-06

**Authors:** Violetta C. Spoeler, Markus Kipp, Daniel Dubinski, Joshua D. Bernstock, Artem Rafaelian, Svorad Trnovec, Cajetan I. Lang, Thomas M. Freiman, Sami Ridwan, Friedrich Prall, Florian Gessler, Sae-Yeon Won

**Affiliations:** 1 Department of Neurosurgery, Rostock University Medical Center, 18057 Rostock, Germany; violetta-chiara.spoeler@uni-rostock.de (V.C.S.); daniel.dubinski@med.uni-rostock.de (D.D.); artem.rafaelian@med.uni-rostock.de (A.R.); svorad.trnovec@med.uni-rostock.de (S.T.); thomas.freiman@med.uni-rostock.de (T.M.F.); florian.gessler@med.uni-rostock.de (F.G.); 2 Institute of Anatomy, Rostock University Medical Center, 18057 Rostock, Germany; markus.kipp@med.uni-rostock.de; 3 Department of Neurosurgery, Brigham and Women’s Hospital, Harvard Medical School, Boston, MA 02115, USA; jbernstock@bwh.harvard.edu; 4 Department of Cardiology, University Medical Center Rostock, 18057 Rostock, Germany; cajetanimmanuel.lang@med.uni-rostock.de; 5 Department of Neurosurgery, Klinikum Ibbenbüren, 49477 Ibbenbueren, Germany; 6 Institute of Pathology, Rostock University Medical Center, 18057 Rostock, Germany; friedrich.prall@med.uni-rostock.de

**Keywords:** hydrogen peroxide, H_2_O_2_, neurosurgical survey, mesothelial damage, vacuolization

## Abstract

**Background**: Hydrogen peroxide (H_2_O_2_) is a well-known hemostatic and antiseptic agent in neurosurgical practice. While there are concerns regarding the use of H_2_O_2_ due to its potential for neuronal damage, the pathophysiological effect on neuronal cells is not clearly understood. **Methods**: An online survey concerning the use of H_2_O_2_ was conducted in a board-certified platform, and an experimental study was designed to investigate the effect of H_2_O_2_ on neuronal and tumor cells. Brain tissues of mice and brain/tumor tissues of humans were irrigated with H_2_O_2_ 3%, H_2_O_2_ 1.5%, and NaCl 0.9%, and processed by bipolar coagulation. Tissue sections were obtained and stained with H&E and analyzed by the depth and degree of neuronal damage measured from the cortical surface (μm). **Results**: In total, 242 neurosurgeons participated in the survey, and 81% of neurosurgeons reported use of H_2_O_2_ in neurosurgical practice. however only 5% of the participants had a literature-based knowledge of the pathophysiological mechanism of H_2_O_2_. In total, eight mouse brain tissues, 21 human brain tissues, and seven human tumor tissues were processed and analyzed. The experimental study found that H_2_O_2_ caused vacuolization of neuronal tissue in mouse brain tissues, with a mean depth of damage of 343.7 ± 39.7 μm after 2 min and 460.1 ± 36.4 μm after 10 min exposure to H_2_O_2_ 3% (*p* < 0.001). In human brain tissues, vacuolization was detected in sections exposed to H_2_O_2_ 1.5% and 3%, with a mean depth of damage of 543.8 ± 304.5 μm and 859.0 ± 379 μm (*p* = 0.003). In the bipolar coagulation group, the mean depth of neuronal damage, of 2504 ± 1490 μm, was nearly three times greater than that in the H_2_O_2_ group (*p* < 0.001). Similar results were observed in human tumor tissues as well. **Conclusions**: H_2_O_2_ seems to cause less local damage on neuronal and tumor cells than conventional bipolar cauterization, suggesting it as a good alternative to be used for hemostasis and marginal tumor cell treatment. However, due to its potential risk for embolism, H_2_O_2_ should be used with caution.

## 1. Introduction 

Hydrogen peroxide (H_2_O_2_) has been a staple in the field of neurosurgery for many decades. Its unique properties as a powerful oxidizing agent have made it an indispensable tool in surgical practice, achieving hemostasis and antiseptic effects [1]. In recent years, research has explored the potential of hydrogen peroxide in the treatment of certain brain tumors as well [2,3,4]. Despite its widespread use in neurosurgery, the mechanisms of action of H_2_O_2_ are not yet fully understood, and its use is not without controversy. Some studies have raised concerns about its potential toxicity to neural cells, particularly when used in high concentrations or for prolonged periods [3,5]. Further, there are several published cases in both spinal and cranial surgery reporting vulnerability to gas embolism with a rare case of clinically relevant morbidity [1,3,6,7,8,9]. Due to its two-sided characteristic, there are a variety of individual opinions about hydrogen peroxide in neurosurgical practice. Therefore, the aim of this study was first to perform a survey concerning the use of H_2_O_2_ in neurosurgery to gain clarity regarding current practice. According to the results of the survey, there was significant ambiguity concerning the pathophysiological effects of H_2_O_2_ on neuronal cells, which led us to the second aim of the study, namely, analyzing the penetration depth and damaging effects of H_2_O_2_ on neuronal cells in vitro and in vivo. In this context, the effect of bipolar coagulation as an inevitable hemostasis tool in neurosurgery was analyzed as well.

## 2. Results

### 2.1. Result of Online Survey

**Figure 1:**
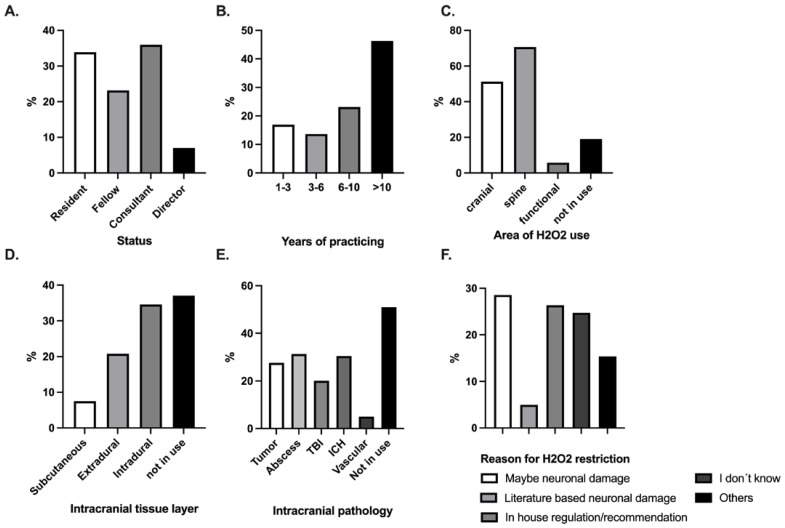
Web-based online survey on the topic “use of hydrogen peroxide in neurosurgical practice”. (**A**) Status in neurosurgical practice. (**B**) Years of neurosurgical practice. (**C**) Surgical area of H_2_O_2_ use. (**D**) Limit of intracranial tissue layer for H_2_O_2_ use. (**E**) Intracranial pathology where H_2_O_2_ is used. (**F**) Reason for restriction of H_2_O_2_ use. H_2_O_2,_ hydrogen peroxide; TBI, traumatic brain injury; ICH, intracerebral hemorrhage.

In total, 242 neurosurgeons practicing throughout Germany participated in the online survey. Of the respondents, 36.0% were consultants, 33.9% residents, 23,1% fellows, and 7% directors, respectively. Nearly half had more than 10 years of experience in neurosurgery. The use of H_2_O_2_ in neurosurgical practice was confirmed by 81% of participants, whereas 19% did not use H_2_O_2_. In particular, 62% of respondents confirmed the use of H_2_O_2_ in intracranial surgery; more than half of those (55.0%) used it up to as deep as the intradural tissue layer. In the case of H_2_O_2 use_ up to intradural tissue, its use was similarly distributed in variety of surgical procedures, including tumor, abscess, traumatic brain injury, and intracerebral surgery. In the case of vascular surgery, the use of H_2_O_2_ was less frequent. The last question focused on reasons for the restrictive use of H_2_O_2_. Among all respondents, 28.6% assumed neuronal injury, 26.4% reported use based on departmental of internal regulations, and 24.7% did not know the reason. Interestingly, only 5.0% of the neurosurgeons had a literature-based knowledge of the pathophysiological mechanism of H_2_O_2_ concerning neuronal damage. Detailed results of survey are shown in Figure 1.

### 2.2. Basic Demographics of the Participants

In total, four patients donated their brain/tumor tissue for the study’s purposes. The mean age of patients that donated brain and tumor tissue was 55.6 ± 6.0 years, and two patients were female (50%). Of the four patients, two (50%) had glioblastoma and the other two had metastasis (50%). None of the patients received a presurgical treatment like radiation or chemotherapy, and the surgical treatment was the first-line therapy according to the interdisciplinary neurooncologic conference.

### 2.3. Hydrogen Peroxide Exposure in Mouse and Human Brain Tissue

In total, eight mouse brain tissue samples, 21 human brain tissue samples, and seven human tumor tissue samples were processed and analyzed. For all slices of mouse and human brain tissues, the occurrence of vacuolization was independent of the status of intact arachnoidal/pial layer or subcortical tissue exposure.

The slices of mouse brain tissue after exposure to NaCl 0.9% and H_2_O_2_ in 1.5% and 3% concentrations divided by different time periods (*n* = 8) are illustrated in Figure S1. There was mesothelial damage visible with the occurrence of vacuolization in brain tissue exposed only to H_2_O_2_ 3%. After 2 min exposure, the mean depth of the damage was 343.7 ± 39.7 μm, whereas after 10 min exposure, the mean depth of damage was significantly higher (460.1 ± 36.4 μm; *p* < 0.0001; diff. 95% CI 75.5–157.3; effect size d_Cohen_ 3.1). In slices of mouse brain tissue after exposure to NaCl 0.9% across different time lines, there was no occurrence of vacuolization at all.

**Figure 2:**
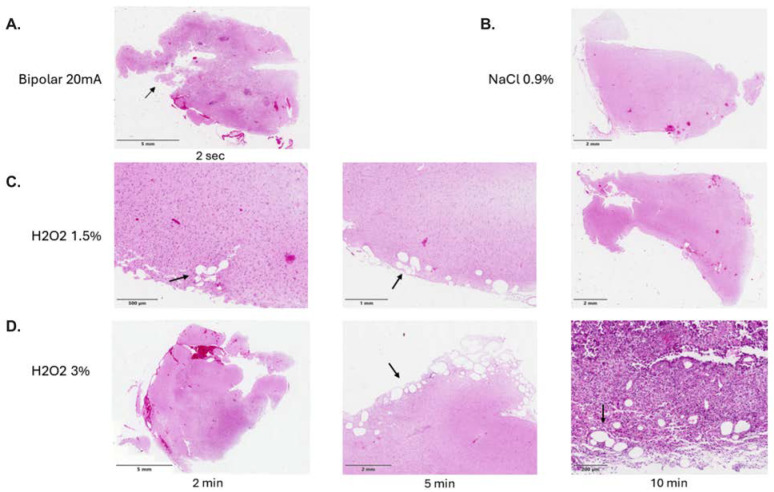
Human healthy brain tissue after exposure to bipolar cauterization (**A**), NaCl 0.9% (**B**), and H_2_O_2_ 1.5%/3% (**C**,**D**). Mesothelial vacuolization as a sign of damage is visible after exposure to H_2_O_2_ (see arrows).

**Figure 3:**
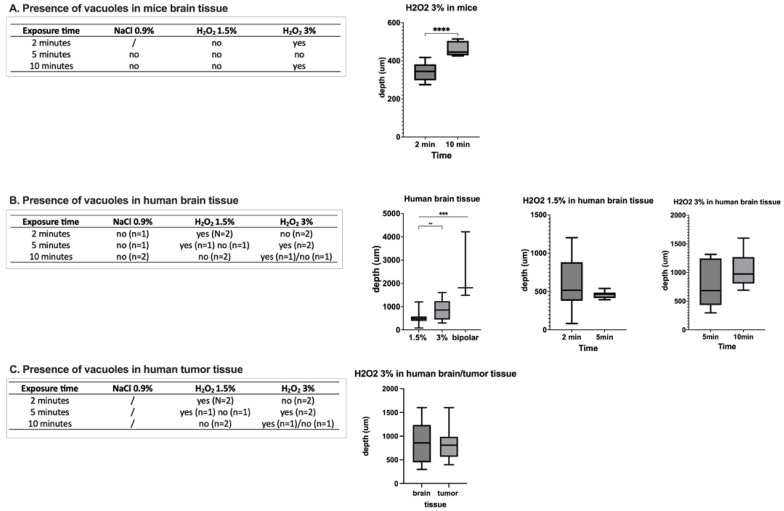
Presence and depth of vacuolization in mouse (**A**) and human healthy (**B**) tumor tissues (**C**). There was a significant difference depending on the concentration of H_2_O_2_ (1.5% versus 3%, *p* < 0.01). ** *p* < 0.01; *** *p* < 0.001; **** *p* < 0.0001.

Slices of human healthy brain tissue after exposure to NaCl and H_2_O_2_ 1.5%/3% and bipolar cauterization with 20 mA (*n* = 19) are illustrated in Figure 2. Vacuolization in human brain tissue could be observed in 50% of the cases after exposure to 1.5% as well as 3% H_2_O_2_. The depth of damage was significantly different among those groups: 543.8 ± 304.5 μm in H_2_O_2_ 1.5%, 859.0 ± 379 μm in H_2_O_2_ 3%, and 2504 ± 1490 μm in the bipolar cauterization group (H_2_O_2_ 1.5% vs. bipolar: *p* < 0.001; diff. 95% CI 1368–2553; effect size d_Cohen_ 1.8; H_2_O_2_ 3% vs. bipolar: *p* < 0.001; diff. 95% CI 994.5–2296; effect size d_Cohen_ 1.5; H_2_O_2_ 1.5% vs. H_2_O_2_ 3%; *p* = 0.003, diff. 95% CI 137.5–492.9; effect size d_Cohen_ 0.9; respectively). Of note, the depth of damage was nearly three times greater in the bipolar cauterization group compared to the H_2_O_2_ groups. Concerning the duration of H_2_O_2_ exposure, there was no significant difference among the groups (Figure 3). Similarly to the mouse brain slices, there was no occurrence of vacuolization or mesothelial damage across different time lines in human brain slices exposed to NaCl 0.9%.

In the case of tumor tissue, vacuolization was only visible in 66.7% of cases in the group with H_2_O_2_ 3% exposure (Figure S2). Comparing the depth of damage between healthy brain tissue and tumor tissue, there was no statistical difference after exposure to H_2_O_2_ 3% (767.9 ± 387.9 μm vs. 1041 ± 300.0 μm; *p* > 0.05; diff. 95% CI −250.4–171; effect size d_Cohen_ 0.8) (Figure 3).

### 2.4. Characterization of Mesothelial Vacuolization in Mouse and Human Brain Tissue

**Figure 4:**
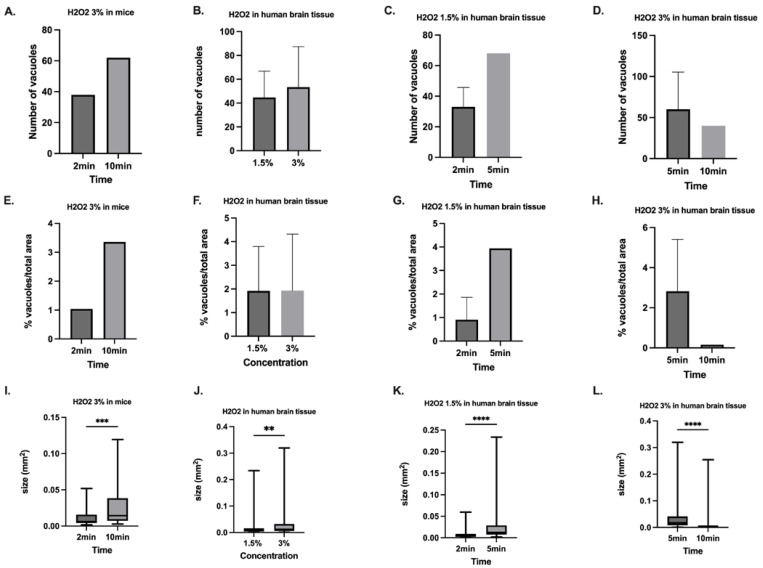
Characterization of vacuoles in mouse and human healthy/tumor brain tissues (**A**–**D**). Number of vacuoles (**E**–**H**), Ratio of vacuoles/total area (**I**–**L**), Mean size of the vacuoles after processing with H_2_O_2_ in mouse and human brain tissues depending on the concentration and duration of exposure. The total size of vacuoles was significantly larger after longer exposure to H_2_O_2_ 3% in mouse brain tissues (0.01 ± 0.01 mm^2^ versus 0.03 ± 0.03 mm^2^, *p* < 0.001). In human brain tissues, the sizes of vacuoles were significantly larger in H_2_O_2_ 3% compared to H_2_O_2_ 1.5% (0.02 ± 0.03 mm^2^ versus 0.03 ± 0.06 mm^2^; *p* = 0.001). After longer exposure, significantly larger sizes were detected in human brain tissues exposed to H_2_O_2_ 1.5% (*p* < 0.001), whereas the results were inconsistent in human brain tissue exposed to H_2_O_2_ 3%. ** *p* < 0.01; *** *p* < 0.0001; **** *p* < 0.00001.

The number of vacuoles, percentage of vacuole areas, and the total size of the vacuoles are illustrated in Figure 4.

In mouse brain tissues, the number of vacuoles, the percentage of vacuole areas, and the total size of vacuoles were higher after longer exposure to H_2_O_2_ (*n* = 38 versus 62; 1.0% versus 3.4%; 0.01 ± 0.01 mm^2^ versus 0.03 ± 0.03 mm^2^, *p* < 0.001; diff. 95% CI 0.006–0.024; effect size d_Cohen_ 0.9).

In human brain tissues, the results were inconsistent. The number of vacuoles and vacuole sizes in terms of area were not different between the different concentrations of H_2_O_2_; however, the sizes of vacuoles were significantly larger in tissues exposed to H_2_O_2_ 3% compared to H_2_O_2_ 1.5% (0.02 ± 0.03 mm^2^ versus 0.03 ± 0.06 mm^2^; *p* = 0.001; diff. 95% CI 0.004–0.025; effect size d_Cohen_ 0.2). There was a trend toward higher numbers and larger vacuole areas after longer exposure to H_2_O_2_ 1.5%; however, surprisingly, the numbers of vacuoles and their areas were lower and smaller, respectively, after longer exposure to 3% H_2_O_2_.

## 3. Discussion

In this study, we investigated the pathophysiological effects of H_2_O_2_ in neuronal tissues with a focus on its use as a potential hemostatic and tumor treating tool in neurosurgery. Our results showed that neuronal damage induced by H_2_O_2_ was dependent on concentration and duration and independent of intact arachnoidal/pial layer. The depth of neuronal damage was limited to up to 1 mm, which was 2.5 times less than that caused by bipolar coagulation. The effect was visible both in healthy brain and tumor tissues.

To date, H_2_O_2_ has been continuously used in a variety of different fields, including plastic, orthopedic, and general surgery, as well as neurosurgery [1]. The primary concern surrounding the use of H_2_O_2_ in neurosurgery is its potential neurotoxicity, as evidenced by the current survey, which revealed that 20% of respondents do not utilize it, and when it is used, it is more frequently employed in spinal surgery. However, when asked about their reason for H_2_O_2_ restriction, less than 5% of respondents could support it with literature-based evidence; therefore, we felt a responsibility to pursue this matter with a preclinical and clinical study.

The first scientific study to evaluate the in vitro effects of H_2_O_2_ on neuronal tissue was conducted by Hansson and Vallfors, published in the 1980s [10]. Therein, there was thrombosis of leptomeningeal vessels and extensive blood–brain barrier dysfunction, with cell disintegration observed by exposure to H_2_O_2_ 3%. In a more recent study, by Mesiwala et al., the damage to the surface was limited to the arachnoid surface, and up to 1 mm of tissue beyond the resection margin was characterized by vacuolization and degeneration of neurons, astrocytes, and microglia [3]. Our study was able to show results similar to those of the previous study, with development of stomal vacuolization of up to 1 mm, whereas the lower concentration of H_2_O_2_ (1.5%) showed significantly less invasiveness, with smaller vacuole sizes and numbers. Otherwise, the duration of exposure was not a significant factor for increased damage in human brain tissue compared to mouse brain tissue. Of note, bipolar coagulation showed significantly increased invasive damage to the neuronal cells and connective tissue compared to the use of H_2_O_2_.

There have been several H_2_O_2_-related cases reported with adverse effects [2,5,6,7,8,9]. The most dreaded complication is gas embolism in the vessel, which might cause, albeit rarely, severe ischemic and systematic sequalae. This effect underlies the extremely efficient decomposition of H_2_O_2_ into water and O_2_ by exposure to the family of catalase enzymes (200,000 reactions per second), which are found in all cells [11]. Usually, the cascade is physiological to avoid the production of cell-damaging hydroxyl radicals. The microbubbles induce mechanical removal of tissue debris, thus exhibiting antimicrobial properties [12]. On the other hand, 1 mL of H_2_O_2_ 3% produces 10 mL of O_2_. In the case of an excess amount of H_2_O_2_, the sudden high O_2_ burden can result in oxygen embolization, with systemic embolism in coronary or cerebral arteries [5,13]. Further, H_2_O_2_ has the property of inducing vasoconstriction, whereas there are some inconsistencies depending on the vascular segment and species as well [12]. The risk of gas embolism related to the use of H_2_O_2_ is not restricted to intracranial procedures, but occurs also in other types of surgery as well, such as in spinal, traumatic, and orthopedic procedures. In a recent review, some risk factors for vulnerability to gas embolism were identified, like open dural wounds with close relationships to major vessels and the seated position of the patients [1]. In our clinical experience of using H_2_O_2_ in over 50 cranial surgeries (not illustrated in this study), all postoperative MRI scans performed within 3 months after surgical treatment were evaluated and showed no radiological signs of embolic ischemic complications nor abnormalities in the diffusion-weighted sequence in the resection margin of the brain tumors or intracerebral hemorrhage. Of note, we do not perform intracranial surgery with patients in the seated position anymore due to the risk of gas embolism independent of the use of H_2_O_2,_ and restrict the use of H_2_O_2_ in closed cavities. In a previous study, there was one case of embolic complication reported in over 800 surgical cases, resulting in a complication rate of 0.1% [12]. While there are still concerns surrounding the use of H_2_O_2_ in neurosurgery, our study suggests that its cautious use (e.g., H_2_O_2_ in a diluted concentration of 1.5% with an exposure time of less than 2 min), with respect to the correct positioning of patients and local application in the resection cavity, might be beneficial for hemostasis in cases of diffuse bleeding or marginal tumor treatment with less local damage compared to the conventional bipolar coagulation and low risk for gas embolism. However, it is again important to be alert to the possibility of gas embolism.

### Limitations

While this study provides valuable insights into the effects of hydrogen peroxide on neural tissue, there are several limitations that should be mentioned.

Firstly, the study only examined the effects of H_2_O_2_ on neural tissue, and did not investigate the effects of other oxidative stressors or antioxidants. Further, the effect of H_2_O_2_ on cellular or molecular areas with its pathophysiological mechanism is still elusive. This limits the generalizability of the findings and highlights the need for further research in this area. Secondly, the study used a relatively small sample size, which may not be representative of the larger population. This may limit the statistical power of the study and increase the risk of type II errors. Finally, the study did not investigate the long-term effects of hydrogen peroxide on neural tissue. In our point of view, there are many confounding factors that might influence the effect on neural tissue like postoperative adjuvant radiochemotherapy, tumor progress, sequalae of traumatic brain injury or intracerebral hemorrhage, or infection, resulting in some limitations to the focus solely on the effect of hydrogen peroxide. However, this is an important area for future research, as the long-term effects of oxidative stress on neural tissue are not well understood. Lastly, the survey was performed using an online CME platform. This might not fully represent the broader neurosurgical community and may suffer from response or selection bias.

## 4. Materials and Methods

### 4.1. Online Survey

An online web-based survey via SurveyMonkey^®^ (SurveyMonnkey Inc., San Mateo, CA, USA) was distributed among participants and newsletter subscribers (*n* = 4500) of an online CME platform “Neurosurgery to Go” during a period of 4 weeks. Participants with neurosurgical backgrounds were selected, and the survey was voluntary; consequently, no ethical approval was necessary since no personal data from participants or patients were collected. The survey consisted of 6 different questions concentrating on the clinical use of H_2_O_2_ in neurosurgery (Table S1).

### 4.2. Preclinical Study

#### 4.2.1. Animals

All experimental procedures adhered to the national guidelines governing the ethical care and use of laboratory animals. Throughout the study, female C57BL/6J mice aged 4 months were utilized. Having previously participated in another experimental protocol, these mice underwent surgical induction of myocardial infarction, three positron emission tomography scans, one magnetic resonance tomography scan, and intravenous administration of 100 µg/100 µL of biotinylated Lycopersicon esculentum (Tomato lectin) (Vector Laboratories, Burlingame, CA, USA) prior to sacrifice. Anesthetic agents employed included pentobarbital sodium, isoflurane, and ketamine/xylazine. Before sacrifice, pentobarbital sodium (50 mg/kg body weight) was administered intraperitoneally.

#### 4.2.2. Experiment

**Figure 5:**
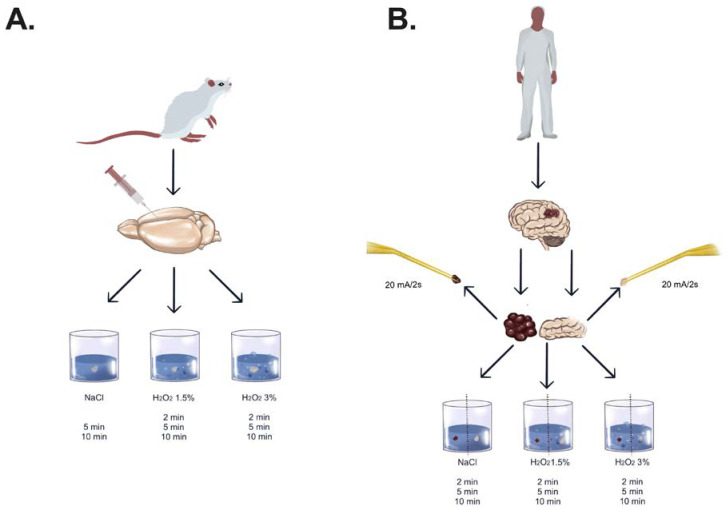
Experimental setting for mouse and human healthy/tumor brain tissue. (**A**) Experimental procedure in mouse brain tissue. After obtaining the brain tissue, a small puncture with 18 gauge needle 2 mm lateral from bregma in the frontal lobe was performed. Afterwards, the tissue was processed with NaCl 0.9%, H_2_O_2_ 1.5%, or H_2_O_2_ 3% with different time frames (2/5/10 min). (**B**) Experimental procedure in human brain tissue. Besides the process with NaCl 0.9%, H_2_O_2_ 1.5%, or H_2_O_2_ 3%, additional brain tissue was treated by bipolar cauterization at 20 mA.

Following sacrifice, decapitation was performed, and the cranium was carefully dissected to extract the intact brain. Subsequently, a small puncture was performed 2 mm from the bregma in the frontal cortex using an 18-gauge needle in order to observe the different reaction in case of intact arachnoidal/pial layer versus subcortical exposure. The brain was immersed in a receptacle containing either H_2_O_2_ 3% (*n* = 3), H_2_O_2_ 1.5% (*n* = 3; prepared by diluting H_2_O_2_ 3% with an equal volume of distilled water) or NaCl 0.9% (*n* = 2), ensuring complete coverage of all surfaces. Following exposure durations of 2, 5, or 10 min, all specimens were retrieved, rinsed with NaCl 0.9% solution (excluding those from the NaCl group), and then transferred to individual containers filled with paraformaldehyde solution 4% (Figure 5A).

#### 4.2.3. Histology

The specimens underwent standard laboratory processing procedures and were embedded in paraffin following established protocols [14]. For sectioning, a rotary microtome (Epredia™ HM 340E) (Epredia, NH, USA) was employed. The resulting sections were then transferred to a water bath (Leica HI 1210) (Leica Biosystems, IL, USA) containing distilled water maintained at 47 °C for optimal mounting onto glass slides (Expredia^TM^ SlideMate^TM^ Microscope Slides). Following this, the slides were incubated overnight at 47.9 °C to facilitate drying. Subsequently, the slides were stained using hematoxylin and eosin (Leica ST5020-CV5030 Stainer Integrated Workstation) (Leica Biosystems, IL, USA), and photomicrographs were captured and digitized using a slide scanner (Grundium Ocus 40) (Grundium, Tampere, Finland) for further analysis.

#### 4.2.4. Analysis

(1) Depth of vacuolization

To evaluate the depth of vacuolization, the virtual slide viewing software, ViewPoint (PreciPoint, Garching, Germany), was employed. By observing all vacuoles throughout the slices, three vacuoles exhibiting the deepest penetration were identified, after which their depth was assessed by three independent examiners. The mean value of the three measurements per vacuole was then calculated. Subsequently, utilizing these mean values, the maximum depth of vacuolization within each experimental group was determined (Figure S3).

(2) Quantity and area of vacuoles

For the quantification of vacuoles and the determination of their areas relative to the total brain tissue area on the slide, we utilized a custom Python script (Python Software Foundation, version 3.9.13, retrieved from https://www.python.org) on 1 February 2024. The script enabled us to manually outline the brain tissue and then identify, outline, and count the vacuoles and calculate their area relative to the total area (Figure S4).

(3) Sizes of vacuoles

The size of each vacuole in pixels was calculated with the Python script as well. To establish a correlation with the actual size of the vacuoles, we used the total number of pixels per image and scaled it by the known length in micrometers using ViewPoint. The size of a single pixel in square micrometers was determined and multiplied by the number of pixels per vacuole. For better visualization, these measurements were converted into square millimeters.

### 4.3. Clinical Study

#### 4.3.1. Participants

All experimental procedures adhered to national guidelines and received approval from the local ethical committee at University Rostock Medical Center (A 2021-0266). Prior to surgery, informed consent was obtained from the patients.

Brain tissues in the access route to the tumor were harvested during routine intracranial surgeries performed for the excision of primary brain tumors in adult subjects (*n* = 3). Further, tumor tissue was harvested during routing surgery for the excision of glioblastoma (*n* = 1). Overall, the surgical methodologies remained unaltered during this study.

#### 4.3.2. Experiment

Upon extraction from the brain, the human tissues were immediately sectioned into multiple fragments, depending on their size. These fragments were subsequently introduced into 20 mL syringes containing either H_2_O_2_ 3% (healthy brain tissue: *n* = 8; tumor tissue: *n* = 3), H_2_O_2_ 1.5% (healthy brain tissue: *n* = 7; tumor tissue: *n* = 3; prepared by diluting H_2_O_2_ 3% with an equal volume of distilled water), or NaCl 0.9% (healthy brain tissue: *n* = 4, served as negative control group). They were then allowed to incubate for 2, 5, or 10 min, ensuring complete immersion. Additionally, other fragments underwent thermocoagulation using bipolar coagulation forceps set at 20 mA (healthy brain tissue: *n* = 2; tumor tissue: *n* = 1; served as positive control group), a technique commonly employed in neurosurgical procedures (Figure 5B). Following treatment, all specimens were transferred to individual containers filled with paraformaldehyde solution 4%. Those specimens exposed to H_2_O_2_ were subsequently rinsed with NaCl 0.9% prior to placement. In a single instance, specimens were only introduced to H_2_O_2_ 3% (2 and 10 min) and H_2_O_2_ 1.5% (10 min). Histological processing and measurements were conducted following the same procedures as those applied to animal tissue specimens.

#### 4.3.3. Analysis

Given the less distinct margins of specimens extracted during surgical procedures compared to those of animal brains, we employed ImageJ 1.41. (Rasband, W.S., ImageJ, U.S. National Institutes of Health, Bethesda, MD, USA) prior to analysis with a Python script to enhance the visualization and distinction of vacuoles. Images were converted to an 8-bit format, and the threshold was set to black and white and manually adjusted for best contrast. Analyses with Python were carried out following the same procedure stated above.

#### 4.3.4. Statistic Analysis

GraphPad version 9.4.2. (GraphPad Software, Boston, MA, USA, www.Graphpad.com) accessed on 1 January 2021 was used for the statistical analysis. Data are expressed as percentage or mean ± standard deviation (mean ± SD). Parametric data were analyzed for between-group differences using one-way ANOVA with post hoc Bonferroni testing. Prior to the analysis, all data sets were tested for normality according to the Kolmogorov–Smirnov test. Nonparametric data sets were analyzed for between group differences using Mann–Whitney U test. Further effect sizes were calculated and defined as follows: little to no effect (0.1–0.3), medium effect (0.3–0.5), strong effect (0.5–0.8), extremely strong effect (0.8–1.0). A *p* value of ≤ 0.05 was considered statistically significant.

## 5. Conclusions

This study elucidates the effects of hydrogen peroxide on neuronal tissue and highlights the importance of considering the potential risk of its use in neurosurgery. Compared to the conventional hemostasis by bipolar coagulation, hydrogen peroxide was found to be less aggressive in terms of local neuronal damage, which was limited to 1 mm from the surface. Further, the effect was visible in tumor cells as well, indicating that hydrogen peroxide might be a potential intraoperative treatment tool to treat marginal tumor cells as well. However, due to the risk of collateral injury, hydrogen peroxide should be used with caution.

## Data Availability

The original contributions presented in this study are included in the article/Supplementary Material. Further inquiries can be directed to the corresponding author.

## Supplementary Materials

The following supporting information can be downloaded at: https://www.mdpi.com/article/10.3390/ph18040533/s1, Figure S1. Brain tissue of mice after exposure to NaCl, H_2_O_2_ 1.5%, and H_2_O_2_ 3% for different time periods (2/5/10 min). Figure S2. Human tumor tissue after exposure to H_2_O_2_ 1.5% and H_2_O_2_ 3% for different time periods (2/5/10 min). Figure S3. Representative slice of mouse brain exposed to H_2_O_2_ 3%, measuring maximum depth of vacuole (352.6 μm). Figure S4. Representative slice of mouse brain exposed to H_2_O_2_ 3% after outlining the vacuoles with Python script. Table S1. Online survey of neurosurgeons on the topic “hydrogen peroxide”.
