# Supplementary material for: The Value of Hydrogen Peroxide in Neurosurgery and Its Pathophysiological Effects in Human and Animal Brain Tissues
Source: Pharmaceuticals (Basel). 2025 Apr 6;18(4):533. doi: 10.3390/ph18040533 (PMC12030584; doi:10.3390/ph18040533)
Supplement: Supplementary file 1 [file pharmaceuticals-18-00533-s001.zip › pharmaceuticals-3567047-supplementary.pdf]

Supplementary Files

Supplementary Tables

Table S1. Online Survey among neurosurgeons with the topic “hydrogen peroxide”

**Questions**	**Answers**
What is your status in the neurosurgical department? (Choose one)	-Resident<br>-Fellow<br>-Consultant<br>-Director
How many years have you been practicing neurosurgery? (Choose one)	-1-3 years<br>-3-6 years<br>-6-10 years<br>->10 years
Do you use hydrogen peroxide in neurosurgical operations? (Choose multiple)	-Cranial<br>-Spinal<br>-Functional<br>-hydrogen peroxide is not used
If you use hydrogen peroxide in intracranial procedure, up to which tissue layer are you using it? (Choose one)	-Subcutaneous<br>-Extradural<br>-Intradural<br>-hydrogen peroxide is not used
If you use hydrogen peroxide in intracranial, intradural procedures, in which surgeries do you use it? (Choose multiple)	-Tumor<br>-Abscess<br>-Traumatic brain injury<br>-Intracerebral hemorrhage<br>-Vascular procedures (e.g. AVM, aneurysm, fistula)<br>-hydrogen peroxide is not used
If you do not use hydrogen peroxide in intracranial procedures, what is the reason for it? (Choose one)	-It may case neuronal damage<br>-Neuronal damage is known based on literature<br>-In-house regulation/recommendation<br>-I don´t know<br>-Other reason (comment)

Supplementary Figures

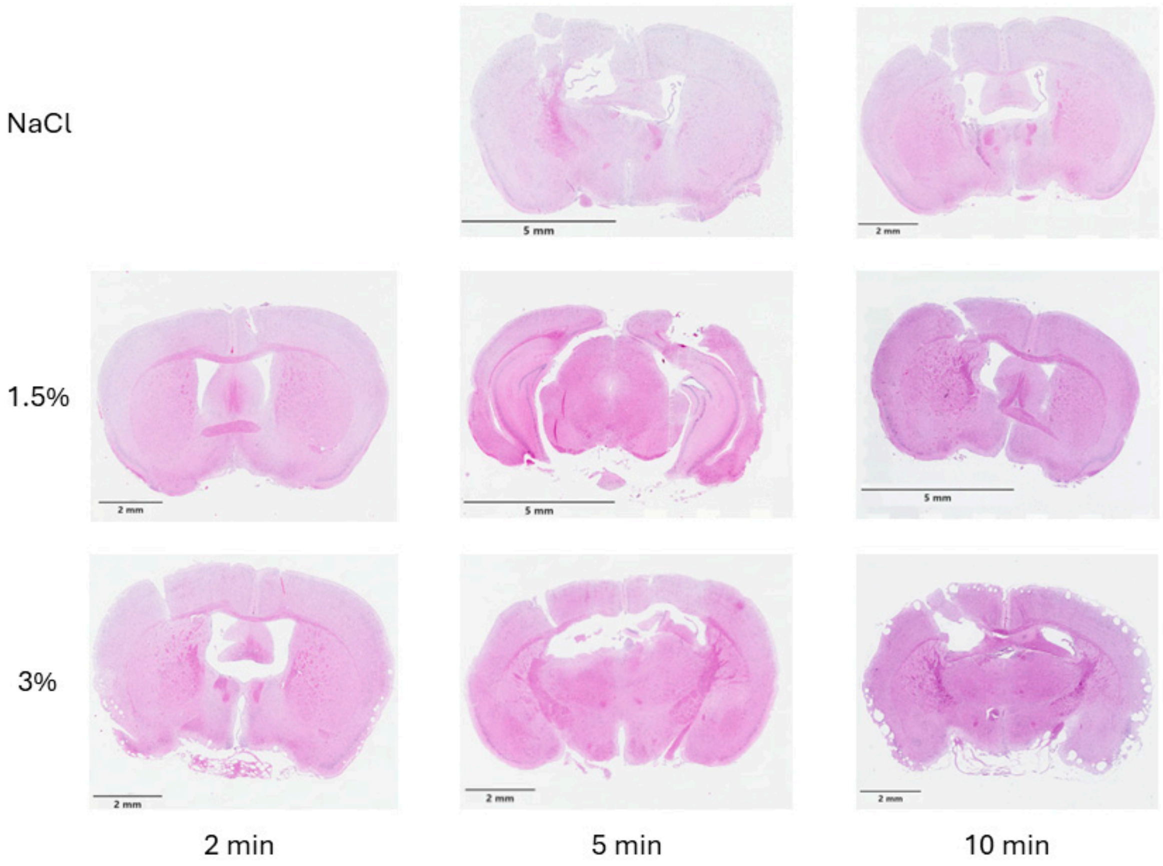

Figure S1. Brain tissue of mice after exposure with NaCl, H_2O_2 1.5% and H_2O_2 3% with different time period (2/5/10 minutes).

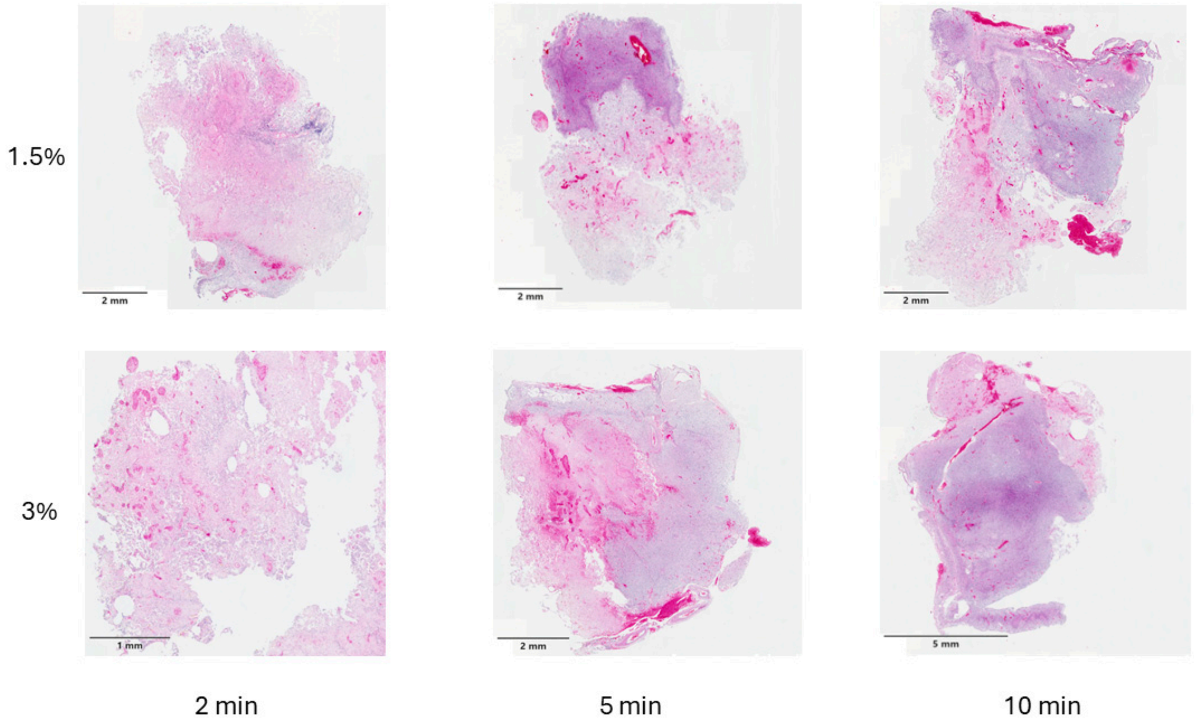

Figure S2. Human tumor tissue after exposure with H_2O_2 1.5% and H_2O_2 3% with different time period (2/5/10 minutes).

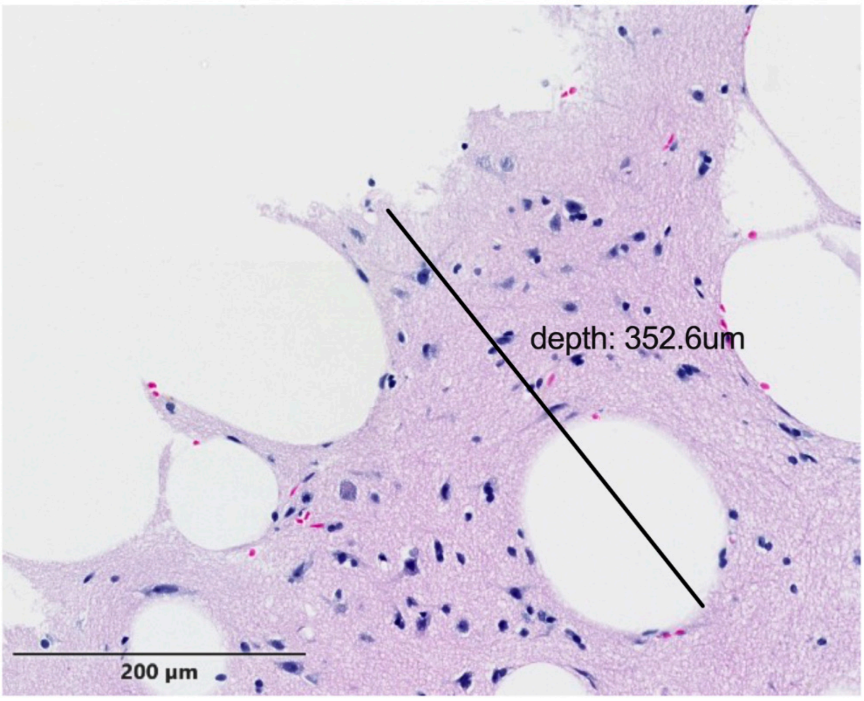

Figure S3. Representative slice of mouse brain exposed with H_2O_2 3% measuring maximum depth of vacuole (352.6 um).

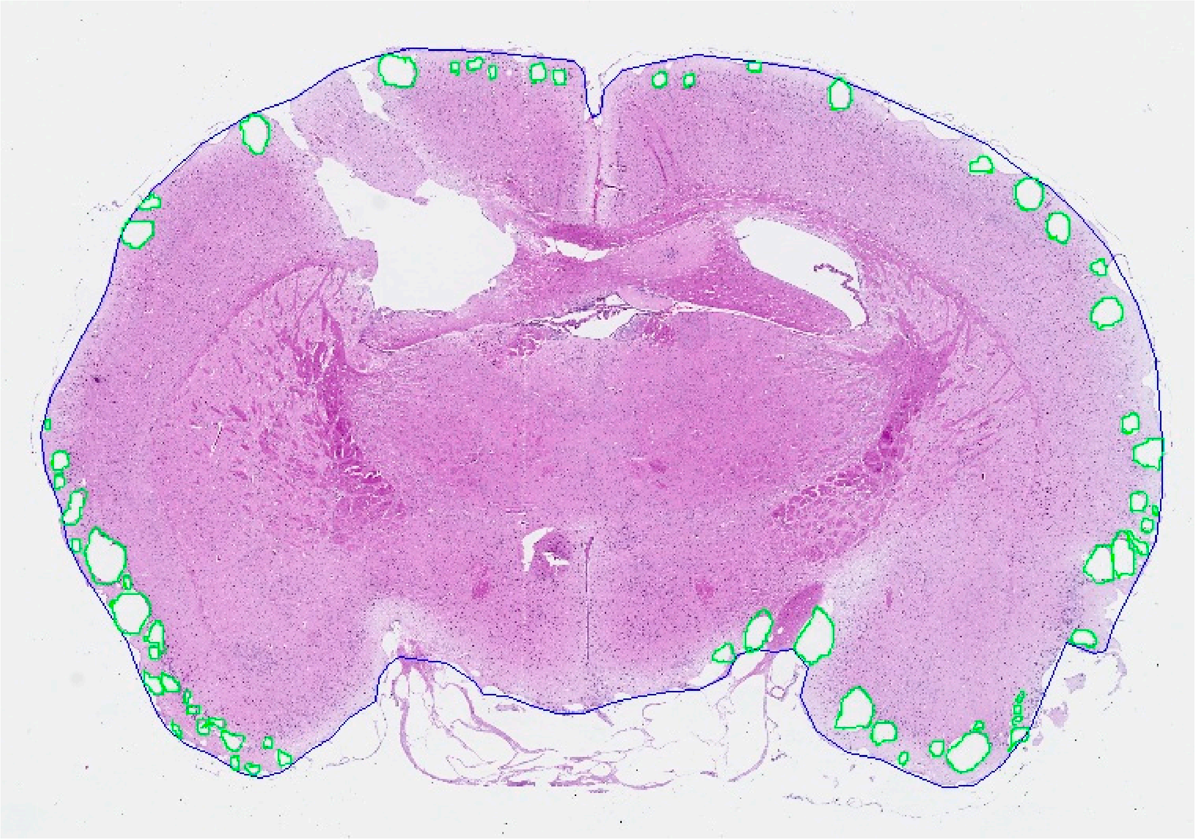

Figure S4. Representative slice of mouse brain exposed with H_2O_2 3% after outlining the vacuoles with Python script.
